# An effective no-reference image quality index prediction with a hybrid Artificial Intelligence approach for denoised MRI images

**DOI:** 10.1186/s12880-024-01387-1

**Published:** 2024-08-12

**Authors:** Prianka Ramachandran Radhabai, Kavitha KVN, Ashok Shanmugam, Agbotiname Lucky Imoize

**Affiliations:** 1 Department of AIML, New Horizon College of Engineering, Bangalore, Karnataka India; 2 Department of Communication Engineering, School of Electronics Engineering, Vellore Institute of Technology, Vellore, Tamil Nadu India; 3 Department of Electronics and Communication Engineering, Vel Tech Multi Tech Dr. Rangarajan Dr. Sakunthala Engineering College, Chennai, Tamil Nadu India; 4 Department of Electrical and Electronics Engineering, Faculty of Engineering, University of Lagos, Akoka, Lagos, 100213 Nigeria

**Keywords:** Image Quality Assessment, Magnetic Resonance Images, Artificial Intelligence, Hybridization, Multi-objective optimization, Performance metrics

## Abstract

As the quantity and significance of digital pictures in the medical industry continue to increase, Image Quality Assessment (IQA) has recently become a prevalent subject in the research community. Due to the wide range of distortions that Magnetic Resonance Images (MRI) can experience and the wide variety of information they contain, No-Reference Image Quality Assessment (NR-IQA) has always been a challenging study issue. In an attempt to address this issue, a novel hybrid Artificial Intelligence (AI) is proposed to analyze NR-IQ in massive MRI data. First, the features from the denoised MRI images are extracted using the gray level run length matrix (GLRLM) and EfficientNet B7 algorithm. Next, the Multi-Objective Reptile Search Algorithm (MRSA) was proposed for optimal feature vector selection. Then, the Self-evolving Deep Belief Fuzzy Neural network (SDBFN) algorithm was proposed for the effective NR-IQ analysis. The implementation of this research is executed using MATLAB software. The simulation results are compared with the various conventional methods in terms of correlation coefficient (PLCC), Root Mean Square Error (RMSE), Spearman Rank Order Correlation Coefficient (SROCC) and Kendall Rank Order Correlation Coefficient (KROCC), and Mean Absolute Error (MAE). In addition, our proposed approach yielded a quality number approximately we achieved significant 20% improvement than existing methods, with the PLCC parameter showing a notable increase compared to current techniques. Moreover, the RMSE number decreased by 12% when compared to existing methods. Graphical representations indicated mean MAE values of 0.02 for MRI knee dataset, 0.09 for MRI brain dataset, and 0.098 for MRI breast dataset, showcasing significantly lower MAE values compared to the baseline models.

## Introduction

In recent years, digital photos, remote sensing, satellite imagery, and medical imaging have all advanced significantly. This growth has resulted in advancements in acquisition systems, including smartphones, digital cameras, and multiple other imaging devices [1]. Therefore, users can now record, store, and share high-resolution images thanks to new acquisition devices. Nevertheless, the blurring, motion artifacts, ambient disruptions, etc., degrade the real-time images [2]. As a result, evaluating an image's clarity is essential and still difficult for academics. An image quality assessment (IQA) algorithm that can correctly assess and forecast the quality of an image similar to the opinions of humans has been developed over the past ten years [3]. The final consumers are given a high-quality encounter by these models. IQA can generally be divided into two groups. The first strategy is a subjective one that relies on human judgments and is very precise. Subjective testing must be integrated into the system as an optimization metric because it is time-consuming and costly [4, 5]. It is optimal to use objective quality measurement techniques for fast system performance evaluation and optimization because they typically use subjective evaluation information as the basis for training. These models naturally forecast the picture quality in connection to how people perceive the images [6].

Subjective evaluation findings are typically the ground truth for teaching objective quality evaluation methods. Under how people perceive things, these models naturally anticipate the image's clarity. The different full-reference (FR) and reduced-reference (RR-IQA) methods have been discussed in the literature, including SSIM [7], PSNR [8], FSIM [9], MSSIM [10], and VIF [11]. However, these methods need to be revised for applications that require real-time processing. This is due to the impossibility of accessing the original image in real time under all imaging circumstances [12, 13].

Therefore, no-reference (NR) methods, also known as blind IQA models, have been the focus of most studies. The quality of an image can be assessed randomly using various criteria. Some of the factors used for NR-IQA include anisotropy [14], wavelet [15], discrete cosine transform (DCT) [16], and Gabor filtering [17]. These methods can evaluate the test picture's quality without previous knowledge of the reference image. Real-time apps are more than insufficient for computational time. In recent years, deep learning has overtaken the fields of computer vision and picture analysis. The application of objective IQA has remained consistent with this pattern. Different deep convolutional networks were used to estimate perceptual picture quality, including VGG16 [18], InceptionV3 [19], ResNet50 [20], DenseNet201 [21], InceptionResNetV2 [22], and NASNetMobile [23]. However, traditional techniques require a lot of computation and take longer to train. Recently, MRI analysis has played an important role in the modern health field. The scanner takes images from different body parts like joints, legs, head, abdomen etc. Due to this reason, the MRI scanners of different images are taken in this research for quality assessment. Consequently, various methods are used to analyze the MRI scanner image quality, but the finest outcomes still need to be achieved [24, 25]. Therefore, a novel intelligent methodology is proposed to estimate the NR-IQA model accurately.

In our study, we have integrated features extracted from EfficientNet-B7 into our Non-Reference Image Quality Assessment (NR-IQA) framework to significantly enhance the accuracy and robustness of image quality evaluation. EfficientNet-B7, renowned for its exceptional performance in image classification tasks, offers a valuable resource of hierarchical feature representations acquired from a wide spectrum of images. These features are particularly beneficial for NR-IQA applications as they enable the extraction of intricate details and semantic information across different scales, essential for comprehensive image quality assessment.By incorporating EfficientNet-B7 features, our methodology gains several advantages. Firstly, it excels in hierarchical feature extraction, capturing detailed nuances and semantic context crucial for assessing image quality comprehensively. This capability is pivotal in discerning perceptually important characteristics from less relevant ones, thereby enhancing the precision and depth of our quality assessment framework. Moreover, leveraging transfer learning with pre-trained weights from EfficientNet-B7 empowers our NR-IQA model to generalize effectively across diverse image types, even in scenarios where reference data is limited. This transfer learning capability not only enhances the model's adaptability but also contributes to its stability and reliability across varying image qualities and conditions. The discriminative power of EfficientNet-B7 features further elevates our approach by enabling more accurate differentiation between different levels of image quality. This enhanced discriminative ability ensures that our framework maintains robust performance consistency, crucial for its practical application in real-world scenarios. Overall, by integrating EfficientNet-B7 features, our methodology represents a significant advancement in NR-IQA. Beyond improving accuracy, it establishes a scalable solution capable of handling diverse image datasets with greater precision and reliability. This integration underscores the potential of leveraging advanced classification models to enrich image quality assessment methodologies, setting a foundation for more nuanced and effective evaluations in the field of image processing.

The following are the significant contributions of the study:

- The proposed bilateral incorporated Wiener filter enhances the Wiener filter by incorporating bilateral filtering, which preserves edge information in MRI images. This improves image quality and reliability for clinical diagnosis and research, surpassing the performance of traditional Wiener filtering techniques.
- The work introduces the GLRLM and EfficientNet B7 algorithms for extracting features from denoised MRI images. GLRLM analyzes texture by characterizing consecutive pixel runs, while EfficientNet B7 is a deep learning model efficient in image classification. Together, they enable effective analysis and feature extraction for improved MRI interpretation.
- The Multi-Objective Reptile Search Algorithm (MRSA) is introduced for selecting the best feature vector. MRSA is designed to handle multiple objectives simultaneously, making it suitable for feature selection tasks where multiple criteria need to be optimized. By using MRSA, the proposed method can effectively identify the most relevant features from a given feature set, thereby improving the efficiency and effectiveness of the feature selection process.
- The self-evolving deep belief fuzzy neural network (SDBFN) method is recommended for a successful study of NR-IQ. SDBFN combines the capabilities of deep belief networks and fuzzy logic to effectively model and analyze non-reference image quality (NR-IQ). This method is particularly suitable for tasks where reference images for comparison are not available, such as in video quality assessment or image enhancement. By using SDBFN, researchers can improve the quality and reliability of NR-IQ studies, leading to more robust and effective image quality assessment techniques.
- The comprehensive evaluation of earlier designs using large IQA datasets as benchmarks is crucial for advancing image processing. It involves testing existing methods across diverse images to identify their strengths and weaknesses, leading to improved algorithms and standardized benchmarking procedures.

The remainder of the article is arranged as follows: A summary of the current approaches is provided in Related work section. Proposed methodology section presents the proposed NR-IQ evaluation methodology. Finally, Result and discussion section presents an overview of all the tests and key findings, and Conclusion section explains the conclusions reached.

## Related work

Many NR-IQA techniques have been written about in journals in recent years. Distinction-specific and general NR-IQA algorithms can be categorized into multiple categories. An NR-IQA model that is rotation-invariant and numerically effective was introduced by Rajevenceltha, J., and Gaidhane [26]. The hyper-smoothing LBP (H-LBP), additionally referred to as the modified LBP and Laplacian of H-LBP (LH-LBP), symbolizes the framework of the image. Support vector regression (SVR) is used in the image quality forecast algorithm to gauge the quality of the image. Varga, D. [23] developed a new, deep learning-based NR-IQA design. It is built on the decision merging of numerous image quality ratings from various convolutional neural networks. This method's primary premise is that various networks can describe real image distortions more accurately than an individual network. Furthermore, relevance and cross-database analyses have supported these findings.

Bagade, J.V. et al. [27] suggested a hybrid method built on machine learning for assessing NR quality. The feed-forward neural network is given information in the form of blockiness-based parameters, additional statistical parameters, and NSS-based characteristics. The quality value is predicted by the backpropagation training method. This number and the differential mean opinion score are linked. (DMOS). The above factors are also used as classification data for support vector machines. This classifier's measured success is 89%. Obuchowicz R. et al. [28] presented a new BIQA technique for assessing MR images. It has been found that the effectiveness of non-maximum suppression (NMS) filtering is highly influenced by the perceived quality of the incoming picture. The entropy of a series of extrema numbers determined with the thresholded NMS effectively describes the quality. The introduced measure works significantly better than similar techniques by a wide margin because it corresponds with human scores better, according to the comprehensive experimental assessment of the BIQA methods.

The Self-evolving Deep Belief Fuzzy Neural Network (SDBFN) integrates deep belief networks (DBNs) and fuzzy neural networks (FNNs), combining deep learning's hierarchical data representation with fuzzy logic's handling of uncertainties and non-linearities. This hybrid approach excels in modeling complex relationships within image data for Image Quality Assessment (IQA), capturing both high-level semantic features and low-level perceptual details crucial for accurate evaluations. Recent studies have demonstrated SDBFN's superiority over traditional methods in IQA tasks, especially in non-reference scenarios, due to its adaptive learning and robust feature extraction capabilities. Its integration of fuzzy logic enhances its ability to address subjective aspects of image quality perception, improving metric accuracy and interpretability. Overall, SDBFN represents a significant advancement in IQA, promising more reliable and comprehensive image quality evaluation methodologies.

When employing image quality statistics, a hybrid deep neural network (DNN) is suggested to record image features related to image quality; this method ensures that important image features can be used to forecast image quality. Chan, K.Y., et al. [29]. Additionally, a tree-based classifier called geometric semantic genetic programming is suggested to carry out the general forecasts by fusing CNN predictions and image characteristics. Although, at the same time, this method is more straightforward than completely connected networks, it is still capable of modeling nonlinear image qualities. A deep design with a region proposal network (RPN) for blind natural-scene and screen-content-based image quality evaluation, known as DeepRPN-BIQA, was suggested by ur Rehman, M. et al. [30]. Critical areas are recovered by moving the network across the extracted feature map from deep networks, such as VGGNet and ResNet, using the texture and borders of the images. The area of interest comprises all regions suggested (RP) that share more than 60% of their boundaries. (ROI). Each ROI receives a local quality score, and the overall quality score is calculated by averaging all of the native quality values.

A neural network-based BIQA utilizing two-sided pseudo reference (TSPR) images was introduced by Hu, J., Wang, X, et al*.* [31]. Following the extraction of the bilateral distance plots between the TSPR pictures and the original warped images, the regression neural network models how the human brain interprets information. The experiment findings show the algorithm's effectiveness and reliability, providing better performance than that of cutting-edge NR techniques.

A study by [35] presents a new diffeomorphism-based approach for non-rigid medical image registration across modalities like MRI, CT, and 3D rotational angiography, using a non-stationary velocity field and a similarity energy function. The researchers invested in optimizing this framework to address deformation challenges and demonstrated its promising quality, with MSE of 1.3136, NCC of 0.9962, SS of 0.9897, MI of 0.883, FSIM of 0.9922, and MAE of 1.52 ± 2.09, evaluated on both private and public datasets [35]. Tracking instruments in laparoscopic and robotic surgeries can enhance focus and reduce errors, but remains challenging due to motion blur, noise, lack of texture, and occlusion, with existing methods being time-consuming and less accurate for high-volume data [36]. This paper presents a semi-automatic algorithm to enhance contrast in low-contrast MRI and reconstruct the left ventricle surface using a new graph cut method, showing promising results with an average Dice coefficient of 92.4% and a Hausdorff distance of 2.94 mm [37]. This work introduces a comprehensive pipeline for cerebral blood flow simulation and real-time visualization, addressing the critical clinical challenges in accurate detection and effective therapy for cerebral aneurysms [38]. This article presents a real-time, cost-efficient platform for HemeLB simulation and visualization of cerebral aneurysms, with the Jetson TX1 outperforming the Zynq SoC by a factor of 19 in site updates per second [39]. This paper presents efficient hardware architectures for lattice Boltzmann simulations on a Zynq SoC, achieving a 52-fold speedup over a dual-core ARM processor implementation [40]. Several studies have advanced medical image segmentation techniques using innovative neural network architectures and methodologies. ConvUNeXt, incorporating ConvNeXt-inspired features into UNet, demonstrated enhanced segmentation performance with reduced parameters [41]. Res-PAC-UNet focused on liver CT segmentation, achieving high accuracy using Pyramid Atrous Convolutions and a fixed-width residual UNet backbone, showcasing a Dice similarity coefficient of 0.958 ± 0.015 with minimal parameters [42]. Another study proposed an efficient encoder-decoder DCNN model integrating ResNet and DenseNet features, surpassing existing methods in accuracy while maintaining fewer parameters [43]. For ultrasound image segmentation, a neural network-based approach using Pyramid Scene Parsing emphasized noise removal, achieving a Dice coefficient of 0.913 ± 0.024 and real-time processing capability of 37 frames per second [44]. CoTr introduced a novel framework combining CNNs with an efficient Deformable Transformer for 3D medical image segmentation, significantly enhancing performance on complex datasets [45]. Segmentation methodologies for hepatocellular carcinoma imaging were categorized by their clinical utility in surgical and radiological interventions, highlighting their impact on diagnosis and treatment outcomes [46]. Additionally, the impact of CADe/CADx systems on post-hepatic resection patient health was studied through simulations varying tumor characteristics, providing insights into their potential clinical benefits [47]. A systematic review on immediate post-ablation response in malignant hepatic tumors using fusion imaging systems evaluated clinical outcomes and technical metrics, contributing valuable insights into treatment efficacy [48]. Deep learning techniques for ultrasound image segmentation over the past five years were critically reviewed, focusing on neural network architectures tailored for handling low-contrast and blurry ultrasound images [49]. Lastly, GAN methodologies for synthesizing elastograms from B-mode ultrasound images were reviewed, emphasizing improvements in diagnostic quality and discussing challenges for pocket ultrasound applications [50].

Previous research shows that computational algorithms, such as KNN, SVC, and MLP, can effectively predict drug permeability across the placental barrier with high quality, providing an alternative to animal testing [51, 52]. Research also shows that the tree-based ensemble models like random forest and extra trees, combined with mol2vec fingerprints and SMOTE, achieve high quality in predicting blood–brain barrier permeability for drug repurposing in neurological diseases [53]. Reviews [54] shows that methodologies for estimating age and gender from ECG data, highlighting that elevated ECG age is linked to cardiovascular diseases and mortality, and discusses improvements and clinical applications of these estimations. This study introduces the MEFood dataset for Middle Eastern food recognition, benchmarks various computer vision models, and finds EfficientNet-V2 to excel in performance and resource efficiency, while providing comprehensive analysis and insights [55]. This study evaluates neural networks for ECG-derived age estimation, addressing ECG acquisition parameters, ethnic diversity, and signal distortions, finding that fine-tuning pre-trained networks and employing random cropping schemes enhance performance and reduce data requirements.

The summary of the literature is explained in Table 1.

**Table 1:** Summary of related works

References	Datasets	Methods	Metrics	Merits and limitations
Rajevenceltha, J. and Gaidhane [26]	LIVE database	H-LBP and LH-LBP, SVR (LHL-IQA)	PLCC, SROCC, and RMSE	• High correlation coefficient • Lack of prediction analysis of huge data
Varga, D., [23]	CLIVE, KonIQ-10k, SPAQ and TID2013	DF-CNN-IQA	PLCC and SROCC	• A large number of data is considered • High training time
Bagade, J.V et al. [27]	LIVE database	DMOS-SVM	Correlation coefficient and error	• Less computational complexity • Poor accuracy
Obuchowicz, R et al. [29]	MRI images	ENMIQA	SRCC, correlation coefficients, PLCC, Kendall and root mean square error f	• Computational complexity is very small • A small amount of data is considered for the evaluation
Chan, K.Y et al. [30]	TID2013 database	DNNGP	PLCC, F-test, t-test	• High confidence level • Fewer performance metrics evaluation
ur Rehman, M et al. [31]	Natural scene and screen content	DeepRPN-BIQA	Correlation	• Effective performance achieved for massive data • Lack of performance analysis
Hu, J., Wang, X et al. [29]	LIVE-2D, TID2013, and CSIQ	Deep Network	PLCC and SROCC	• High prediction accuracy • Due to various distortions, the classifier's training is complicated

The literature study reveals that the aforementioned methods address individual bias. Images may include multiple anomalies. The quality of an image cannot be determined by looking at a single artifact because the picture may contain a variety of blur, blockiness, and buzzing artifacts. It is necessary to establish a basic framework.

## Proposed methodology

**Fig. 1:**
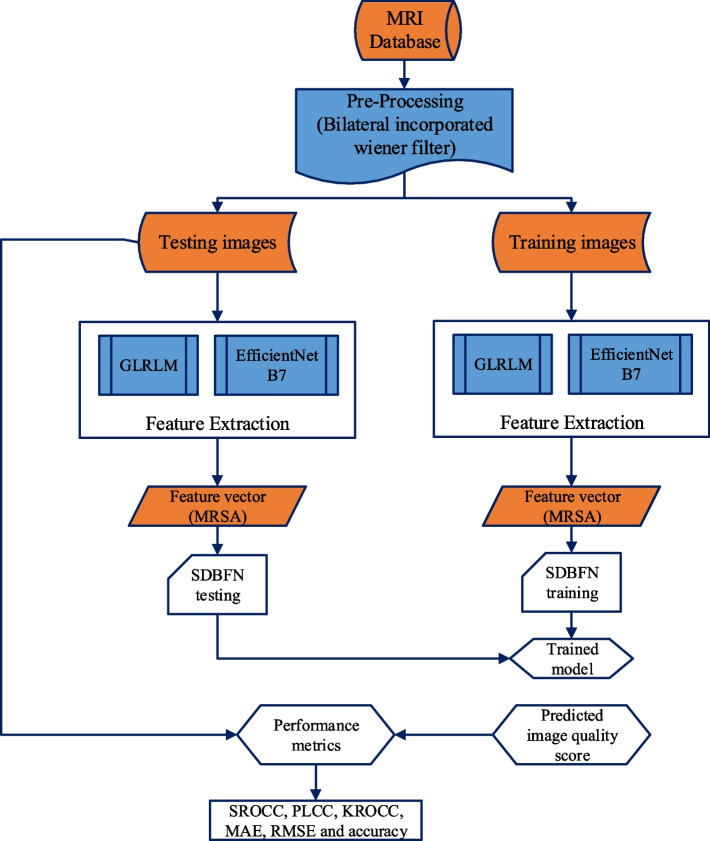
Proposed methodology

This article proposes a hybrid AI method for predicting image characteristics that combines optimization with neuro-fuzzy assessments and image features extracted from distortion measures. The proposed strategy is highly inspirational for incorporating quality measures into hybrid AI and handling the visual comprehension of poor-quality images. This suggested technique also paves the way for categorizing the performance of both high- and low-quality images. The proposed NR-IQA algorithm's block layout is shown in Fig. 1.

The MRI images are gathered from the public database and filtered the images with varying noise levels using a bilateral incorporated wiener filter. To extract the features from the pre-processed images using GLRLM and EfficientNet B7 algorithms are used. The MRSA is provided on the feature vector that estimates the extraction's optimal features. The total image quality assessment value is calculated, and the image's most important estimated characteristics are added together. Furthermore, SDBFN is applied to analyze the quality of images as low or high. The parameters of SDBFN are optimized using the MRSA algorithm.

### Pre-processing

The pre-processing step is the initial stage for removing the unwanted noise from the input images. The bilateral incorporated wiener filtering is an efficient edge-preserving smoothing technique that softens the image while maintaining the clarity of its borders. It is done by merging the two Gaussian filters. While the second filter works in the intensity domain, the first filter operates in the geographic domain. A weighted total of the input is what this non-linear filter produces as its output. The result of the bilateral filter is explained as follows for a $n$ pixel in (1):

1 $$G(n)=\frac1{K(n)}\sum\nolimits_{r\in\phi}{I_ra(\left\|r-n)b\right\|(\left\|I_r-I_n\right\|})$$

where $K(n)=\sum\nolimits_{r\in\phi}{I_ra(\left\|r-n)b\right\|(\left\|I_r-I_n\right\|})$ is denoted as "normalization". $I$ represents the initial input picture that needs to be filtered; $n$ represents the coordinates of the current pixel that needs to be filtered; A Gaussian function can be used as the range kernel $b$, which smoothes differences in values, and the spatial (or domain) kernel $a$, which smoothes differences in coordinates (this function can be a Gaussian function). Additionally, image evaluation employs the Weiner filter. When the contrast is strong, the filter smooths very little. The filter will flatten the picture more when there is a lot of contrast. Equation (2) is used to describe how the Weiner filter works

2 $$W(r,n) = f(r,n)\left( {\frac{P(r,n)}{{P(r,n) + \sigma^{2} }}} \right)$$

where $\sigma^2=\frac1{{}_S2}\sum_{r=1}^S\sum_{n=1}^S{}_a2\left(r,n\right)-\frac1{{}_S2}\sum_{r=1}^S\sum_{n=1}^Sf\left(r,n\right)$. One may determine the noise activity's power spectrum density by using the Fourier series to analyze the noise synchrony $P(r,n)$. These combine filters perform better than other image-enhancing filters.

### Feature extraction

In this section, the GLRLM and EfficientNet B7 is used to extract the features from the pre-processed image. Moreover, the contrast, edge, sharpness and other significant features are extracted by the EfficientNet B7 algorithm. The selection of six features from the Gray-Level Run Length Matrix (GLRLM) based on their direct relevance to the task at hand, such as image classification or quality assessment, ensuring they capture essential aspects of image content. These features are chosen for their high discriminative power, effectively differentiating between various textures or patterns in images and thereby enhancing the accuracy and robustness of the analysis. The selection also prioritizes computational efficiency by limiting the feature set to six, ensuring feasibility within practical time constraints while minimizing redundancy and potential overfitting.

#### GLRLM features

The statistic of interest in the Gray Level Run Length Matrix (GLRLM) is the number of combinations of gray level values and their duration of lines in a particular Region of Interest (ROI). Only seven GLRLM characteristics, known as the Short Run Emphasis (SRE), Low Gray Level Run Emphasis (LGLRE), Long Run Emphasis (LRE), Run Length Non-Uniformity (RLN), Gray Level Non-Uniformity (GLN), Run Percentage (RP), and High Gray Level Run Emphasis (HGRE), will be extracted in this study. The features are explained as follows in (3)-(9):

3 $$SRE = \sum\limits_{{j \in M_{h} }} {\sum\limits_{{k \in M_{s} }} {\frac{{Q_{jk} }}{{k^{2} }}} } /\sum\limits_{{j \in M_{h} }} {\sum\limits_{{k \in M_{s} }} {Q_{jk} } }$$

4 $$LGLRE = \sum\limits_{{j \in M_{h} }} {\sum\limits_{{k \in M_{s} }} {\frac{{j^{2} Q_{jk} }}{{k^{2} }}} } /\sum\limits_{{j \in M_{h} }} {\sum\limits_{{k \in M_{s} }} {Q_{jk} } }$$

5 $$LRE = \sum\limits_{{j \in M_{h} }} {\sum\limits_{{k \in M_{s} }} {j^{2} Q_{jk} } } /\sum\limits_{{j \in M_{h} }} {\sum\limits_{{k \in M_{s} }} {Q_{jk} } }$$

6 $$RLN = \sum\limits_{{k \in M_{h} }} {\left( {\sum\limits_{{j \in M_{s} }} {Q_{jk} } } \right)}^{2} /\sum\limits_{{j \in M_{h} }} {\sum\limits_{{k \in M_{s} }} {Q_{jk} } }$$

7 $$GLN = \sum\limits_{{j \in M_{h} }} {\left( {\sum\limits_{{k \in M_{s} }} {Q_{jk} } } \right)}^{2} /\sum\limits_{{j \in M_{h} }} {\sum\limits_{{k \in M_{s} }} {Q_{jk} } }$$

8 $$RP = \sum\limits_{{j \in M_{h} }} {\sum\limits_{{k \in M_{s} }} {Q_{jk} } } /M$$

9 $$HGRE = \sum\limits_{{j \in M_{h} }} {\sum\limits_{{k \in M_{s} }} {j^{2} Q_{jk} } } \sum\limits_{{j \in M_{h} }} {\sum\limits_{{k \in M_{s} }} {Q_{jk} } }$$

According to their appearance and historical growth, there is no issue that all of the characteristics listed above fall into the same group. Therefore, in this piece, we are interested in uniformly extracting these 7 characteristics.

#### EfficientNet B7 features

The EfficientNet B7 is the advanced method of convolutional neural network types. Here, the ordinary Rectifier Linear Unit (ReLu) is replaced by a novel activation function dubbed the Leaky ReLu activation function in the EfficientNet. Instead of defining the ReLU activation function to be 0 for negative input $(y)$ values, we define it as an incredibly small linear component of $y$. Equation (10) provides the solution for this activation function.

10 $$T(j) = \max (0.01\, \times \,j,j)$$

This function returns x if the input is positive, but it only returns a very small amount, 0.01 times x, if the input is negative. Because of this, it also produces negative numbers. This small modification results in the gradient of the left side of the curve having a non-zero number. There wouldn't be any more failed neurons there as a consequence. Finding a matrix to map out the relationships between the various scaling parameters of the baseline network is the first step in the compound scaling approach under a fixed resource constraint. EfficientNet used the MBConv bottleneck, a crucial building block first introduced in MobileNet V2, but it did so much more frequently than MobileNetV2 due to its larger "Floating point operations per second" (FLOPS) funding. Blocks in MBConv are composed of a layer that increases and then shrinks the channels, whereas direct links are used between constraints with significantly fewer channels than growth layers. As the layers are designed separately, the computation is slowed down by a ratio of $L_{2}$, where $L$ is the kernel size, which stands for the 2D convolution window's width and height. Equation (11) gives the following mathematical definition of EfficientNet:

11 $$E = \sum\limits_{y = 1,2,3,...n} {C_{y}^{{T_{y} }} } \left( {X\left( {P_{y} ,Q_{y} ,R_{y} } \right)} \right)$$

where $T_{y}$ times in the range of $y$, $C_{y}$ stands for the layer norm. The form input in the tensor of $X$ with respect to the layer x is represented by $\left( {P_{y} ,Q_{y} ,R_{y} } \right)$. The image features switch from 256 × 256 to 224 × 224. The layers have to scale with a proportional ratio adjusted with the following algorithm to increase the model accuracy as in (12)-(13):

12 $$Max_{a,b,c} = Acc\left( {E\left( {a,b,c} \right)} \right)$$

13 $$\begin{aligned}E\left(a,b,c\right)= & \sum_{y=1,2,3,..}C_v^{T_v}\left(X\left(c.P_y,c.Q_y,b.R_y\right)\right)\\\text{Memory}(\mathrm{E}) & <=\text{Defined Memory} \\ \text{FLOPS}(\text{E}) &<=\text{Defined Flops}.\end{aligned}$$

In (12), a, b, and c stand in for height, width, and resolution. Equation (13) displays a number of model levels along with a description of the factors. Further, the features from GLRLM and EfficientNet B7 are combined for best feature selection function.

### MRSA for feature selection

The MRSA is a metaheuristic optimization technique inspired by the natural behavior of reptiles. It operates by simulating the hunting behavior of multiple reptiles in search of prey. In MRSA, a population of candidate solutions, represented as potential prey locations, undergoes iterative improvement through a series of local and global search strategies. Local search mechanisms mimic the movement patterns of individual reptiles exploring their immediate surroundings, aiming to exploit promising regions of the search space. Global search mechanisms emulate collective behaviors such as group hunting or migration, facilitating exploration of diverse regions to avoid local optima. By dynamically balancing exploration and exploitation, MRSA enhances convergence towards optimal solutions across various types of optimization problems. Its effectiveness lies in its ability to adaptively adjust search intensities based on problem characteristics, making it suitable for complex optimization challenges where both precision and robustness are essential.

**Fig. 2:**
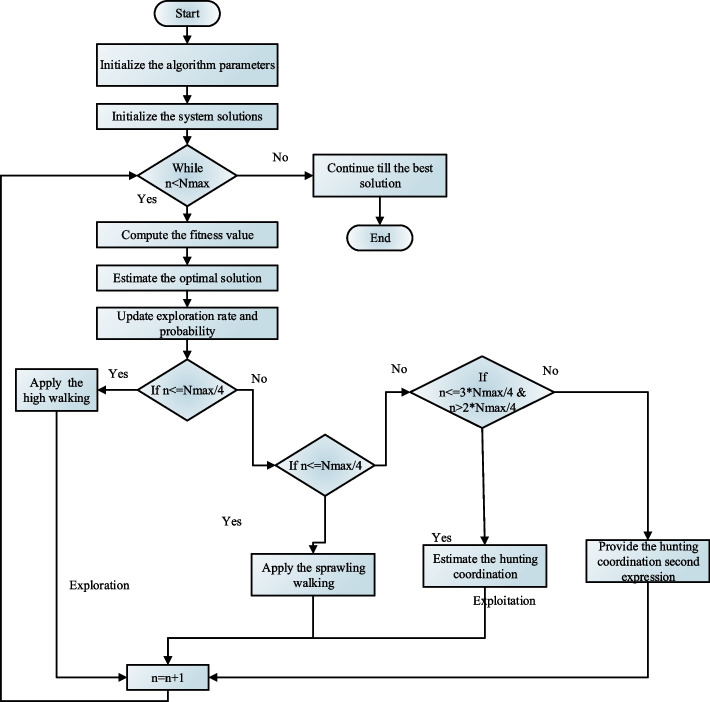
Flowchart of MRSA method

The flowchart of MRSA is illustrated in Fig. 2. The swarm-based optimization method known as the social behavior, foraging tactics, and surrounding style of crocodiles inspired MRSA. The first happens as an encirclement strategy during the investigation stage, and the second occurs as a hunting technique during the exploitation stage. Before the iteration starts, the extracted characteristics are initially applied to the collection of possible answers. It employs an arbitrarily produced strategy given by (14).

14 $$F = \left[ {\begin{array}{*{20}c} {f_{1,1} } & \cdots & {f_{1,k} } & {f_{1,d - 1} } & {f_{1,d} } \\ {f_{2,1} } & \cdots & {f_{2,k} } & \cdots & {f_{2,d} } \\ \vdots & \vdots & \vdots & \vdots & \vdots \\ {f_{M - 1,1} } & \cdots & {f_{M - 1,k} } & \cdots & {f_{M - 1,d} } \\ {f_{M,1} } & \cdots & {f_{M,k} } & {f_{M,d - 1} } & {f_{M,d} } \\ \end{array} } \right]$$

where $d$ represents the crocodile's size, $M$ represents all crocodiles, and $f_{j,k}$ represents the $j^{th}$ crocodile's $k^{th}$ overall area. Equation (14) produces one of many optimum solutions at random given by (15).

15 $$F_{j,k}=lower\,\,P+rand\,\left(Upper\,P-Lower\,P\right)$$

where $Upper\,P$ and $lower\,P$ are the optimization method's upper and lower limits, and $rand$ is an arbitrary integer.

#### Feature exploration

Encircling activity is what sets MRSA's global seeking apart. Crocodiles roam lofty and widespread during the time of the global hunt. The search strategy in MRSA is determined by the amount of active rounds. When $n \le 0.25\,N_{\max }$, MRSA performs an elevated stroll. When $n \le 0.5N_{\max }$ and $n > 0.25TN_{\max }$, the MRSA moves in a spread. These two actions frequently deter crocodiles from pursuing food. The crocodile will eventually stumble upon the broad area of the intended meal, though, as it is a global scan of the complete solved spatial range. In the interim, make sure the amount can be continuously changed to the next developmental stage. The first two-thirds of the overall number of iterations are commonly all that the process lasts. The specific mathematical formulae for the process are described in (16).

16 $$F_{j,k} (n + 1) = \left\{ {\begin{array}{*{20}c} {best_{k} (n) \times \left( { - \delta_{j,k} (n),\,\,\,\,} \right) \times \delta - H_{j,k} (n) \times r,\,\,\,\,\,n \le \frac{{N_{\max } }}{4}} \\ {best_{k} (n) \times f_{{r_{1} ,k}} \times D(n) \times r,\,\,\,\,\,\frac{{N_{\max } }}{4} \le n < \frac{{2N_{\max } }}{4}} \\ \end{array} } \right.$$

where $r$ is a random number between 0 and 1, and $best_{k} (t)$ is the location of the crocodile that is in the best after $n$ repeats. The symbol $N_{\max }$ denotes the utmost repetition. Equation (17) identifies the $j^{th}$ reptile in the $k^{th}$ dimension as the $\delta_{j,k}$ generator. The real wording refers to the sensitive parameter b as 0.1 and governs the search precision.

17 $$\delta_{j,k} = best_{j,k} (n) \times q_{j,k}$$

The reduction function, which is used to reduce the examined $H_{j,k} (n)$ region, is determined using (18).

18 $$H_{j,k} (n) = \frac{{best_{k} (n) - x_{{r_{2} ,k}} }}{{best_{k} (n) + \varepsilon }}$$

where $r_{2}$, and $r_{2}$ is a random number between 1 and M, $x_{{r_{2} ,k}}$ is the $k^{th}$ size of the crocodile at the given position. The randomly declining chance ratio $D(n)$, which has a range of 2 to -2, is contained in (19)

19 $$D(n) = 2 \times r_{3} \times \left( {1 - \frac{1}{N}} \right)$$

where $q_{j,k}$ is the proportion separating the crocodile in the optimal position from those in the present location, updated as in (20).

20 $$q_{j,k} = \alpha + \frac{{best_{k} (n) - M(f_{j} )}}{{best_{k} (n) \times \left( {Upper\,P - Lower\,P} \right) + \varepsilon^{\prime}}}$$

where $M(f_{j} )$ is given in (21) as the crocodile's typical position for $f_{j}$,

21 $$M(f_{j} ) = \frac{1}{t}\sum\limits_{k = 1}^{t} {f_{j,k} }$$

#### Feature exploitation

In this section, the MRSA search phase is linked to the local representational exploitation's hunting process, which has two strategies: cooperation and collaboration. As soon as the process of encirclement kicks in, the crocodiles almost lock in the location of the intended food, and their hunting strategy will make it easier for them to get there. The MRSA conducts when $n < 0.75N_{\max }$ and $n \ge 0.75N_{\max }$ coordinates foraging. When, $n < N_{\max }$ and $n \ge 0.75N_{\max }$ the MRSA uses a joint foraging strategy given by (22).

22 $$F_{j,k} (n + 1) = \left\{ {\begin{array}{*{20}c} {best_{k} (n) \times q_{j,k} (n) \times s,\frac{{2N_{\max } }}{4} \le n \le \,\frac{{3N_{\max } }}{4}} \\ {best_{k} (n) - \delta_{j,k} (n) \times \varepsilon - H_{j,k} (n) \times s,\,\,\,\,\,\frac{{3N_{\max } }}{4} \le n < \frac{{4N_{\max } }}{4}} \\ \end{array} } \right.$$

where $best_{k} (n)$ is the crocodile's preferred position, and $\delta_{j,k}$ is the $j^{th}$ crocodile's supervisor in the $k^{th}$ dimension. With acting $H_{j,k} (n)$ as the reduction function, the expression is used to shrink the region under investigation. Before choosing a new search strategy, MRSA generates the beginning population at random in the search space based on the number of repetitions. The flowchart of the MRSA method is shown in Fig. 2.

### SDBFN algorithm

**Fig. 3:**
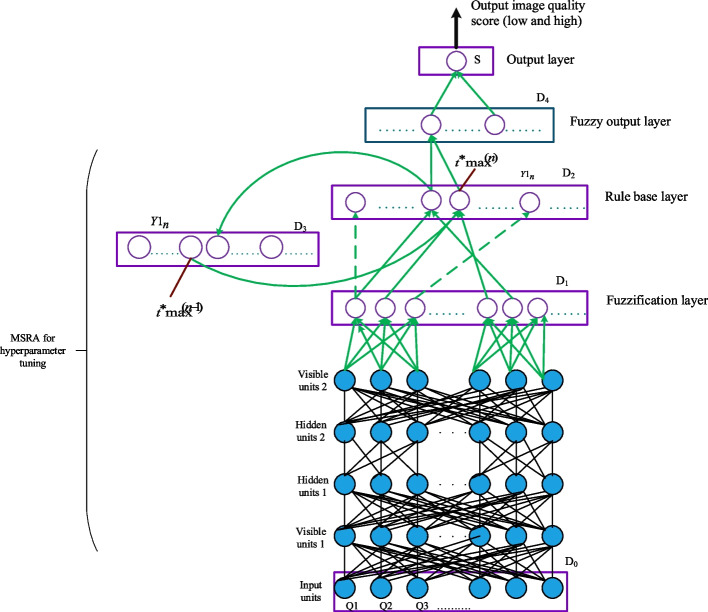
Architecture of proposed MRSA based SDBFN model

The best features selected from MRSA are given to the SDBFN algorithm. The proposed SDBFN method combines a deep belief network and a fuzzy learning approach. The architecture of the proposed MRSA-based SDBFN model is illustrated in Fig. 3. This model is enclosed with the five-layer system, such as the input layer, fuzzification, rule layer, membership function, and defuzzification. The input layer is the first layer. It sends the incoming data variable to the next stage, where it will be fuzzified. Let $Q_{m}$ be the vector of input image features. Equation (23) expresses the input and output of the weight vectors.

23 $$D_1(t^\ast r)=Q_{mf^\ast}\;\mathrm{and}\;D_2(t^\ast r)=S_{mf^\ast}$$

where, the neuron rule is denoted as $t^{ * } r$. Set the visible units to a training vector at the beginning. Update the hidden units concurrently with the visible units given by (24):

24 $$Ph_{m} = P(h_{m} = 1|X) = \delta (b_{n} + \sum\limits_{n}^{{}} {x_{n} D_{mn} )}$$

where, $\delta$ is the logistic sigmoid function and $b_{n}$ is the bias of hidden units. Furthermore, update the visible units concurrently with the hidden units using (25),

25 $$Px_{m} = P(x_{m} = 1|H) = \delta (c_{n} + \sum\limits_{n}^{{}} {h_{m} D_{mn} )}$$

where $c_{n}$ is the bias of visible units. This is referred to as the reconstruction step. Given the rebuilt visible units using the same as the concealed portion, update the hidden units concurrently. Execute the weight update using (26),

26 $$\Delta w_{mn} \alpha < x_{n} h{}_{m} >_{data} + \delta < x_{n} h{}_{m} >_{reconstruction}$$

After a deep belief has been trained, the next deep belief is "stacked" on top of it, using the final learned layer as its input. Next, the new visible layer is set up with a training vector, and the existing weights and biases are used to give values to the units in the learned layers. The above process is then used to teach the new deep belief. Until the intended stopping condition is satisfied, this entire procedure is repeated. Finally, the fuzzification layer is named after a group of spatially arranged neurons that form a fuzzy prediction of the variable indicated by any incoming trained variables. Furthermore, the second layer fuzzifies the inbound data before the third layer gathers it. Equation (27) shows how $D_{1} (n)$ computes the normalized fuzzy distance between a new fuzzy instance $Q_{{1f^{ * } }}$ and a previously $n^{th}$ stored pattern.

27 $$e_{n} = \frac{{\left\| {Q_{{1f^{ * } }} - D_{1} (n)\left\| {_{p} } \right.} \right.}}{{\sum\limits_{n = 1}^{i} {\left\| {Q_{{1f^{ * } }} - L1(n)\left\| {_{p} } \right.} \right.} }}$$

where, $p$ is the p-norm. For p-norms, $\left\| v \right\|_{w + c} \le \left\| v \right\|_{w}$ for any $v \in \Re^{n} ,w \ge 1,c \ge 0.$ The activation levels of rule neurons is given by (28),

28 $$Y1_{n} = 1 - e_{n}$$

where,$Y1_{n} ,e_{n} \in [0,1]$. Furthermore, the third layer is the rule base layer, which contains flexible rule nodes. The nodes reflect the Membership functions, which can be modified during the learning process. Two vectors of bonded weights that are corrected by a mixed supervised/unsupervised learning technique describe rule nodes. The default starting number $R_{n}^{ * }$ is 0.3. During each $x$ time increment (see (29) and (30)):

29 $$If\;\mu>\delta,R_n^\ast\;\mathrm{is}\;\mathrm{decreased},\;R_{n}^{ * } (t^{\prime} + x) = (1 + \frac{\mu - \delta }{x})R_{n}^{ * } (n)$$

30 $$f\;\mu<\delta,R_n^\ast\;\mathrm{is}\;\mathrm{increased},\;R_{n}^{ * } (n + x) = (1 + \frac{\mu }{x})R_{n}^{ * } (n)$$

In (29) and (30), the sensitivity level is indicated by $R_{n}^{ * }$ and $\mu$ the number of neurons is denoted by $\mu$. Saturated linear function type, as stated in (31) is used to disseminate the stimulation of the successful neuron.

31 $$Y_{\max } = \left\{ {\begin{array}{*{20}c} {\begin{array}{*{20}c} 0 \\ 1 \\ {Y_{{(t^{ * } \max )}}\, L2} \\ \end{array} } & {\begin{array}{*{20}c} {if\,Y_{{(t^{ * } \max )}}\, D_{2} < 0} \\ {if\,Y_{{(t^{ * } \max )}}\, D_{2} > 1} \\ {otherwise} \\ \end{array} } \\ \end{array} } \right.$$

The fourth layer is also called fuzzy output, and it serves as an example of fuzzy limitations for the output variables. The header is used to verify the data stream in this case. The systems approve the packet if its confidentiality exceeds the cutoff level; otherwise, they refuse it from transmission over the network. The effective neurons' weight vectors $D_{1}$ and $D_{2}$ along with error vectors $\hat{E}_{r}^{ * }$ are changed from (32) to (33)

32 $$D_{1s} (t^{\prime} + 1) = D_{1s} (t^{\prime}) + \kappa_{1} (Q_{m} - D_{1s} )$$

33 $$D_{2s} (t^{\prime} + 1) = D_{2s} (t^{\prime}) + \kappa_{2} Y_{\max } \hat{E}_{r}^{ * }$$

In equations (32) and (33), $\kappa_{1}$ and $\kappa_{2}$ are the fixed learning values. The output layer is also referred to as the fifth layer. The output layer carries out the de-fuzzification procedure and determines the output variable's number value. The following level receives the rule node's maximum action. The suggested MRSA-based SDBFN model is given in Fig. 3.

The hyperparameter (Learning rate, weight, fuzzy rule, etc.) of the SDBFN model is optimized by the MRSA algorithm exploration and exploitation function's fitness function. If the optimal tuning point is achieved, the accurate image quality prediction is observed as a High or Low-quality score.

### Performance metrics

The correlation level between expected and ground-truth ratings is used to assess NR-IQA algorithms. The research frequently uses the Pearson linear correlation coefficient (PLCC), Spearman rank order correlation coefficient (SROCC), Mean Absolute Error (MAE), and Kendall rank order correlation coefficient (KROCC) to describe the correlation intensity. The PLCC is used to compute the correlation between two data. The definition of PLCC between two vectors $p$ and $q$ of the same length $N$ is in (34)

34 $$PLCC(p,q) = \frac{{\sum\nolimits_{k = 1}^{N} {\left( {p_{{_{k} }} - \overline{p}} \right)\left( {q_{k} - \overline{q}} \right)} }}{{\sqrt {\sum\nolimits_{k = 1}^{N} {\left( {p_{{_{k} }} - \overline{p}} \right)^{2} \sum\nolimits_{k = 1}^{N} {\left( {q_{k} - \overline{q}} \right)}^{2} } }^{\prime } }}$$

where the $k^{th}$ part of $p,q$ is indicated by $p_{{_{k} }}$ and $q_{{_{k} }}$ respectively. Furthermore, $\overline{p} = \frac{1}{N}\sum\nolimits_{k = 1}^{N} {p_{k} }$ and $\overline{q} = \frac{1}{N}\sum\nolimits_{k = 1}^{N} {q_{k} }$. SROCC between these vectors is described as (35)

35 $$SROCC(p,q) = 1 - \frac{{6\sum\nolimits_{k = 1}^{N} {S_{k}^{2} } }}{{N\left( {N^{2} - 1} \right)^{\prime } }}$$

where $S_{k}$ denotes the disparity between each rank's two counterparts $p_{{_{k} }}$ and $q_{{_{k} }}$. Furthermore, KROCC is calculated using the following method defined by (36).

36 $$KROCC(p,q) = \frac{X - Y}{{\left( {\begin{array}{*{20}c} N \\ 2 \\ \end{array} } \right)}}$$

where $Y$ is the number of inconsistent pairs and $X$ is the number of pairs that are in equilibrium with $p_{{_{k} }}$ and $q_{{_{k} }}$. Additionally, the MAE and RMSE technique is the most popular error estimation measure. Using (37) and (38), respectively, the RMSE and MAE number are assessed.

37 $$RMSE = \sqrt {\sum\nolimits_{k}^{N} {\frac{{\left[ {q_{k} - {\text{p}}_{{\text{k}}} } \right]^{2} }}{N}} }$$

38 $$MAE = \sum\nolimits_{k}^{N} {\left| {\frac{{q_{k} - {\text{p}}_{{\text{k}}} }}{N}} \right|}$$

## Result and discussion

The effectiveness of the proposed NR-IQA model is evaluated in this part using data from the MRI databases for the knee, breast, and brain. The MATLAB R2019a system is used for all of the tests, and the system requirements are 16 GB RAM and an Intel(R) Core (TM) i7-9750 CPU operating at 2.6 GHz.

### Dataset description

The MRI knee [32], MRIbrain [33], and MRI breast [34] databases are used in this research for NR-IQA analysis. Training, testing, and validation are conducted to examine the suggested method's effectiveness on the MRI datasets. A 75% training portion, a 15% confirmation portion, and a 10% testing portion make up the collection. The performance study of the suggested model on the training, validation, and test dataset is shown in Table 2. To adjust the SDBFN hyperparameters with the MRSA technique, the proposed NR-IQA model is trained using the training dataset and evaluated on the validation dataset. The learned SDBFN model is also put to the test on the test dataset to determine how effectively it fits.

**Table 2:** Analysis of the proposed NR-IQA's performance on the dataset

MRI database	Overall images	Training	Testing	Validation
Knee	25,000	18,750	3750	2500
Brain	7022	5266.5	1053.3	702.2
Breast	628	471	94.2	62.8

### Performance comparison

**Fig. 4:**
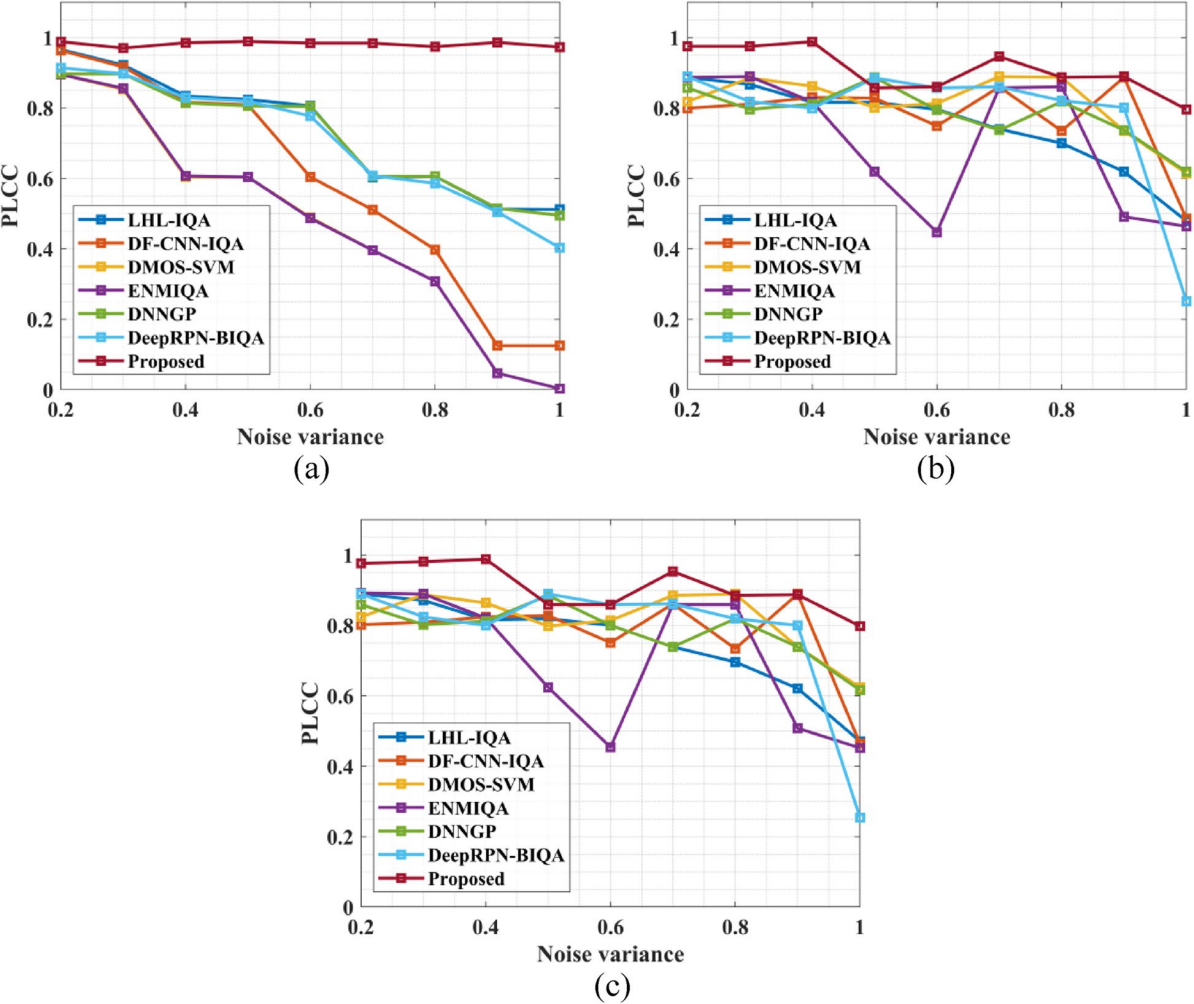
PLCC value (**a**) MRI knee Dataset, (**b**) MRI brain Dataset and (**c**) MRI breast Dataset

The quality of the image metric for a given image is provided by the quality prediction algorithm. The predicted values are contrasted with the mean actual score values to assess the suggested model. The proposed method's and NR-IQA model's effectiveness is assessed using performance measures like SROCC, PLCC, Accuracy, MAE, KROCC, and RMSE. All these performance measures that evaluate the IQA model's effectiveness are computed using the predicted score and mean actual score. The developed model is more precise and more in line with human views, as evidenced by the greater correlation between the projected and mean observed scores. A strong IQA model should have a high correlation and a small mean absolute error with the mean measured score for the predicted quality score. The expected quality score is more closely related to the mean measured score depending on how close the correlation coefficient (SROCC and PLCC) is to 1. Furthermore, the most effective strategy RMSE number ought to be nearer 0. In the present investigation, the suggested model's performance is evaluated in comparison to the performance of the currently available NR-IQA models, including LHL-IQA [24], DF-CNN-IQA [25], DMOS-SVM [26], ENMIQA [23], DNNGP [27], and DeepRPN-BIQA [28]. Figure 4 gives the PLCC value a) MRI knee Dataset, b) MRI brain Dataset and c) MRI breast Dataset.

**Fig. 5:**
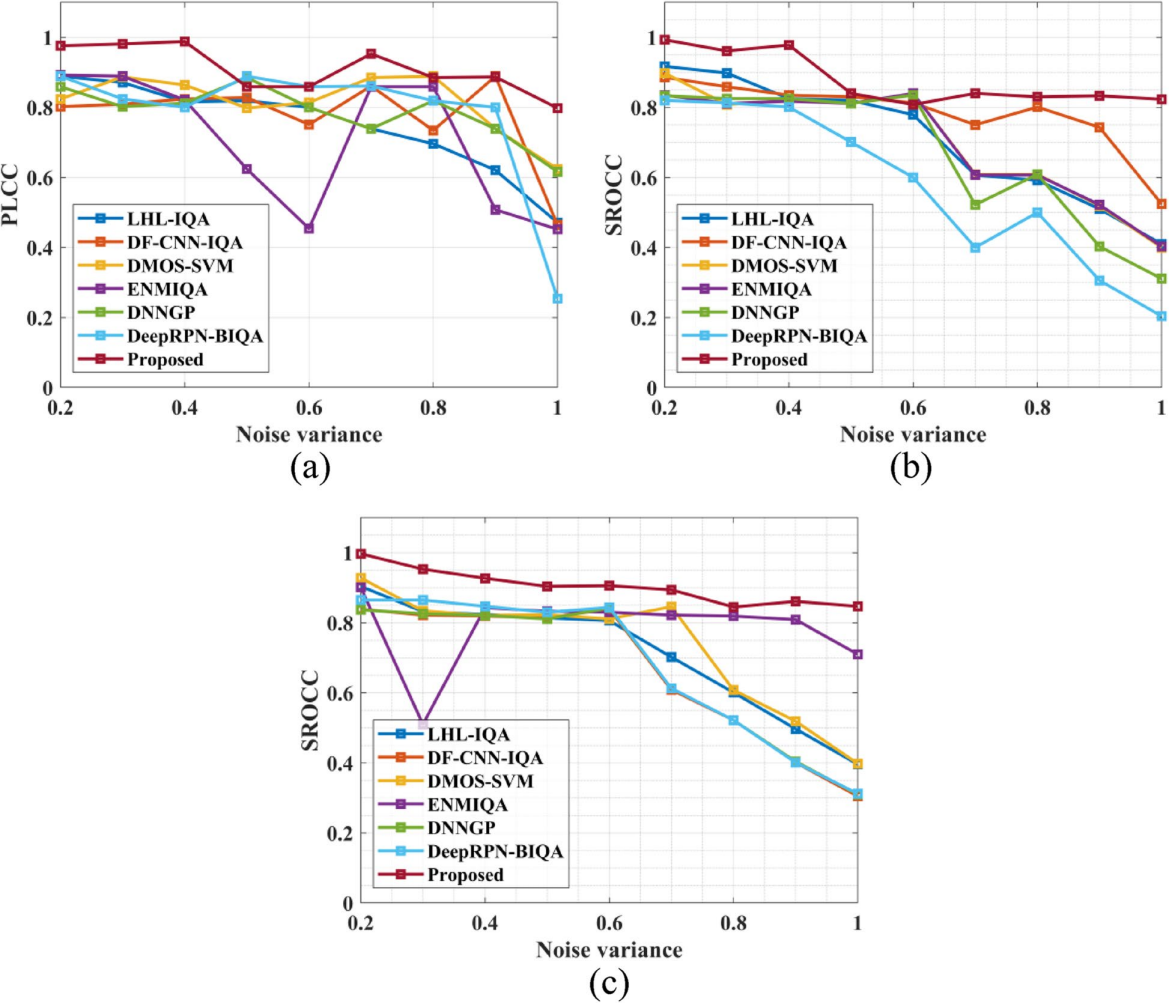
SROCC value (**a**) MRI knee Dataset, (**b**) MRI brain Dataset and (**c**) MRI breast Dataset

The comparison in Fig. 4 between the suggested method and existing models for various noise circumstances reveals compelling insights into the effectiveness of the proposed Non-Reference Image Quality Assessment (NR-IQA) method for MRI images. The graphical representation illustrates that the proposed method consistently outperforms conventional methods across different noise variances. Specifically, the proposed method achieves remarkable average Pearson Linear Correlation Coefficient (PLCC) values of 0.99 for the MRI knee dataset, 0.98 for the MRI brain dataset, and 0.981 for the MRI breast dataset across noise variances ranging from 0.2 to 1.0. In contrast, conventional methods demonstrate lower PLCC values for all noise variances. These results underscore the superior performance of the proposed NR-IQA method in accurately assessing image quality in MRI images, highlighting its potential for enhancing diagnostic and analytical processes in medical imaging applications. Figure 5 presents the SROCC value a) MRI knee Dataset, b) MRI brain Dataset and c) MRI breast Dataset.

**Fig. 6:**
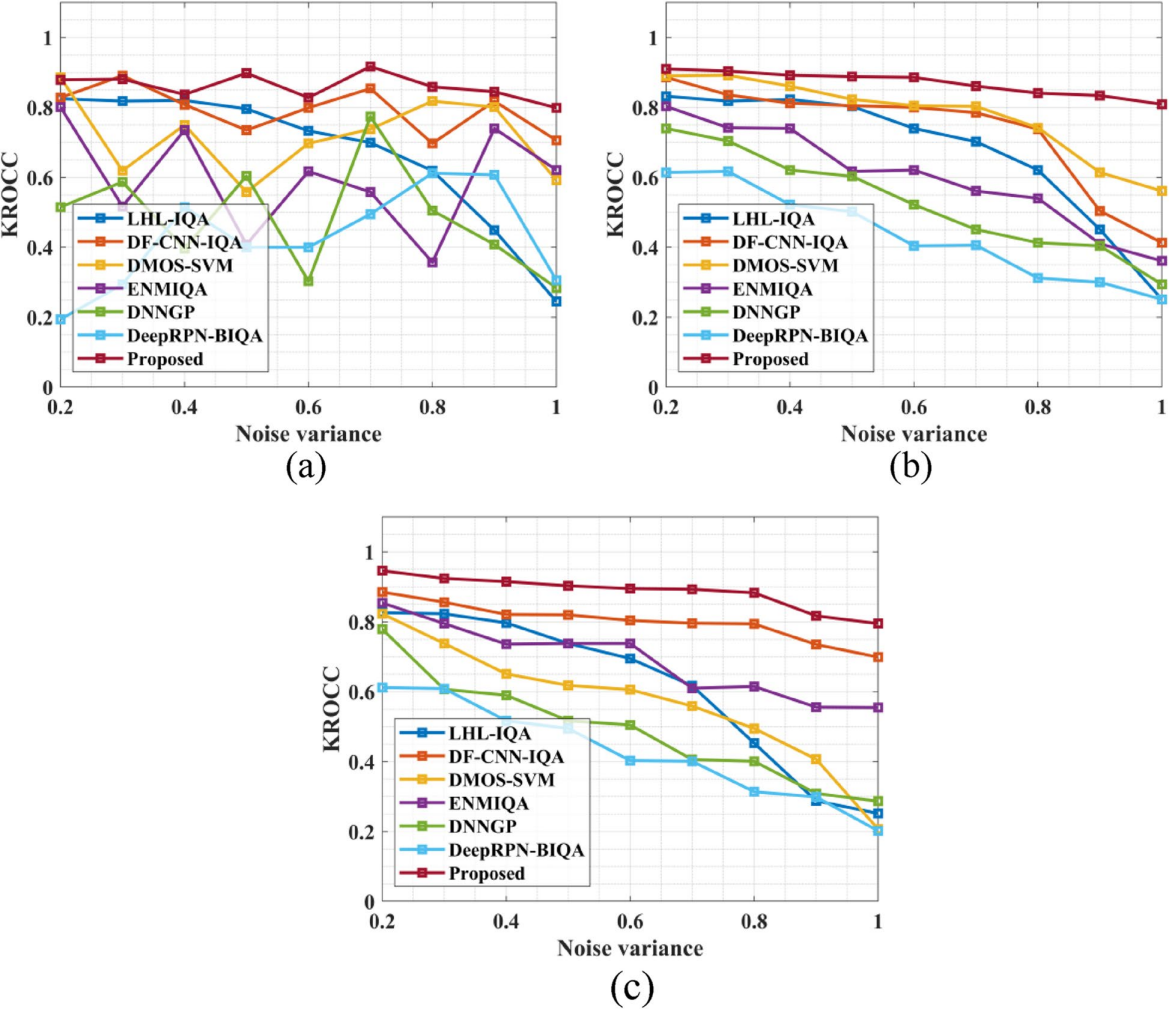
KROCC value (**a**) MRI knee Dataset (**b**) MRI brain Dataset and (**c**) MRI breast Dataset

The analysis extends to the Spearman Rank Order Correlation Coefficient (SROCC) in Fig. 5, which further emphasizes the superiority of the proposed strategy over existing Image Quality Assessment (IQA) methods, particularly in the context of MRI images. The graphical representation reveals that the suggested approach consistently outperforms traditional techniques across varying noise levels. Specifically, the proposed method achieves impressive average SROCC values of 0.991 for the MRI knee dataset, 0.993 for the MRI brain dataset, and 0.981 for the MRI breast dataset at noise levels ranging from 0.2 to 1.0. In contrast, traditional methods yield lower SROCC values for all noise variances. These findings corroborate the overall superior performance of the proposed NR-IQA method, as evidenced by its higher PLCC and SROCC values compared to existing IQA approaches. The results indicate that the proposed method exhibits a stronger correlation with human perception of image quality and demonstrates greater robustness in assessing image quality in the presence of noise, highlighting its potential for enhancing medical imaging applications. Figure 6 shows the KROCC value a) MRI knee Dataset, b) MRI brain Dataset and c) MRI breast Dataset.

**Fig. 7:**
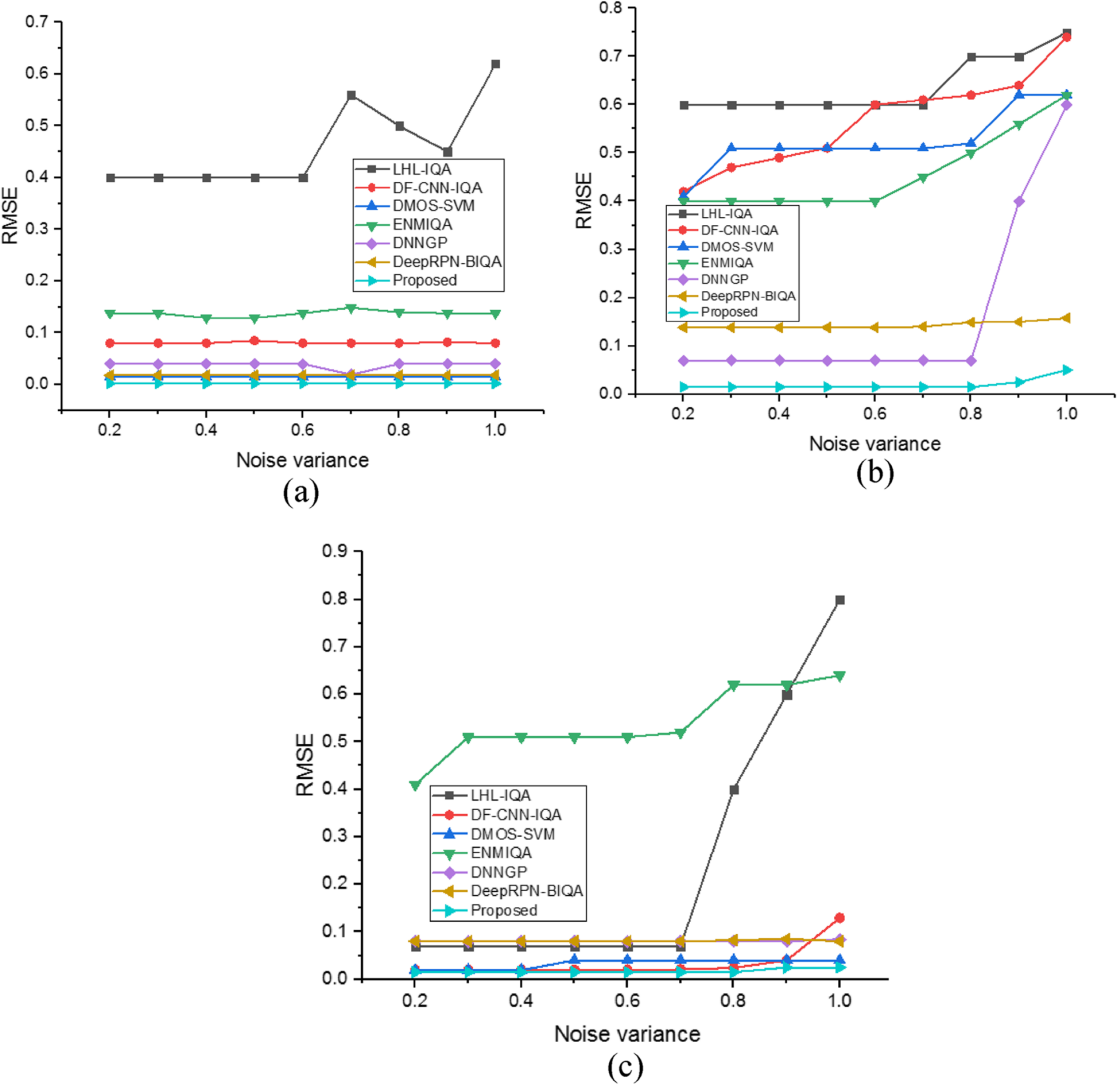
RMSE value (**a**) MRI knee Dataset, (**b**) MRI brain Dataset and (**c**) MRI breast Dataset

Figure 6 presents a comparison of the Kendall Rank Order Correlation Coefficient (KROCC) between the proposed method and traditional techniques across various noise levels, providing further evidence of the proposed method's superior performance in Image Quality Assessment (IQA) for MRI images. The graphical representation indicates that the proposed method consistently outperforms traditional techniques across different noise levels. Specifically, the proposed method achieves mean KROCC values of 0.989 for the MRI knee dataset, 0.91 for the MRI brain dataset, and 0.95 for the MRI breast dataset at noise levels ranging from 0.2 to 1.0. In contrast, conventional methods yield lower KROCC values for all noise variations. These results demonstrate the robustness and reliability of the proposed NR-IQA method in assessing image quality in MRI images, highlighting its potential to enhance diagnostic and analytical processes in medical imaging applications. Figure 7 presents the RMSE value a) MRI knee Dataset, b) MRI brain Dataset and c) MRI breast Dataset.

**Fig. 8:**
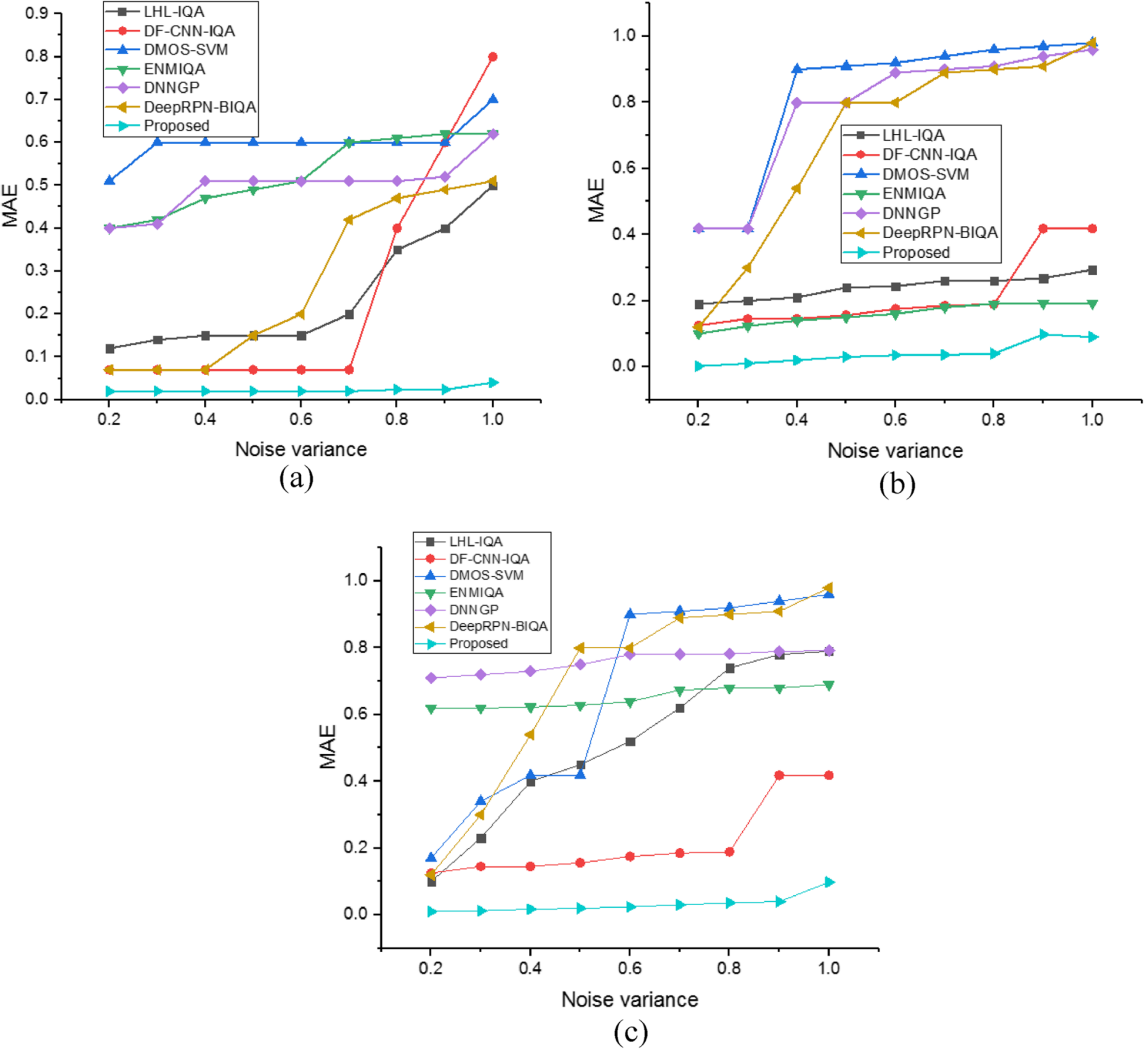
MAE value (**a**) MRI knee Datase, (**b**) MRI brain Dataset and (**c**) MRI breast Dataset

The RMSE number for the proposed model and the existing approaches are displayed in Fig. 7. The graphical depiction shows that the proposed strategy has mean RMSE values of 0.0021 for the MRI knee dataset, 0.015 for the MRI brain dataset, and 0.025 for the MRI breast dataset at noise levels between 0.2 and 1.0. This satisfies the requirements for a successful NR-IQA model. The proposed model, therefore, beats the other systems. It should be noted that the proposed method can locate different kinds of noise associated with images. This shows that the extracted texture and structural data are the main characteristics used to differentiate between high-quality and distorted pictures. This combination of extracted features produces an exact, high-quality figure that closely matches how people view things. The RMSE values are positive and zero. Generally, a lower RMSE value improves system performance. Figure 8 gives the MAE value a) MRI knee Dataset, b) MRI brain Dataset and c) MRI breast Dataset.

The MAE value obtained from the proposed approach compared with the existing methods at altering noise level is illustrated in Fig. 8. The graphical representation demonstrates that, for the MRI knee dataset, 0.02 mean MAE values, for the MRI brain dataset, 0.09 mean MAE values, and for the MRI breast dataset, 0.098 mean MAE values are obtained for the suggested approach at noise levels between 0.2 and 1.0. The analysis shows that the proposed method has achieved significantly less MAE value than the earlier models. The conventional LHL-IQA [24], DF-CNN-IQA [25], DMOS-SVM [26], ENMIQA [23], DNNGP [27], and DeepRPN-BIQA [28] method has obtained high error value than the developed model. This shows the adequate performance of the proposed method in the NR-IQA function. The statistical analysis is found in this research. However, the quality of MRI scanner images are improved with the hybrid filter method and classify the quality of images as high or low with the effective performance. A summary of the proposed method performance is shown in Table 3.

**Table 3:** Summary of proposed method performance

Metric	Proposed Method Performance	Existing Models Performance
PLCC	Achieves average PLCC of 0.99 (knee), 0.98 (brain), 0.981 (breast)	Lower PLCC values for all noise variances
SROCC	Averages SROCC of 0.991 (knee), 0.993 (brain), 0.981 (breast)	Lower SROCC values for all noise variances
KROCC	Mean KROCC of 0.989 (knee), 0.91 (brain), 0.95 (breast)	Lower KROCC values for all noise variations
RMSE	Mean RMSE of 0.0021 (knee), 0.015 (brain), 0.025 (breast)	Higher RMSE values for all noise variances
MAE	Mean MAE of 0.02 (knee), 0.09 (brain), 0.098 (breast)	Higher MAE values for all noise variances

### Discussions

Our research presents a novel approach to No-Reference Image Quality Assessment (NR-IQ) in MRI images by integrating several established techniques to create a robust and efficient framework. By combining the Gray Level Run Length Matrix (GLRLM) and EfficientNet-B7 for feature extraction with the Multi-Objective Reptile Search Algorithm (MRSA) for optimal feature selection, and employing the Self-evolving Deep Belief Fuzzy Neural Network (SDBFN) for analysis, we leverage the complementary strengths of these methods. This integration not only addresses the limitations of existing methods but also achieves significant improvements in performance metrics, including a 20% enhancement in the Pearson Linear Correlation Coefficient (PLCC) and a 12% reduction in Root Mean Square Error (RMSE). Our approach also demonstrates effectiveness across various MRI datasets, with mean Mean Absolute Error (MAE) values of 0.02 for the MRI knee dataset, 0.09 for the MRI brain dataset, and 0.098 for the MRI breast dataset. This comprehensive methodology sets a new benchmark in NR-IQ assessment, highlighting its potential for advancing the field of MRI image quality analysis.

## Conclusion

This article proposes a hybrid approach based on NR-IQA with an optimization method, leveraging artificial intelligence. Without a reference image, this hybrid combo enhances the clarity of any particular medical image. Using a hybrid mix of the suggested systems, the primary goal of this article is to enhance MRI scanner image quality without using a reference image. This study's proposed GLRLM and EfficientNet B7 networks derived features from distorted MRI images by training several images. MRSA primarily increases the quality factor by selecting the most optimal image from the GLRLM and EfficientNet B7 network. With the suggested strategy's help, the system can better distinguish between high- and low-quality images. It also offers high precision, simple computation, versatility, and other benefits. Some of the metrics of the suggested approach, including quality factor, SROCC, PLCC, KROCC, MAE, and RMSE, can be computed using an MRI medical image database. In addition, the quality number is 20% higher than it is under the existing approach. When compared to other techniques currently in use, the PLCC parameter show superior features. When compared to the existing methods, the RMSE number decreased by 12%. Further research would focus on multiple kinds of images and advanced artificial intelligence-based methods for NR-IQA.

## Future scope

Future research could explore enhancing the hybrid AI model by incorporating more advanced deep learning architectures such as transformers or graph neural networks to further improve feature extraction and prediction accuracy. Additionally, investigating the scalability of the proposed model across larger datasets and different types of MRI images could provide insights into its robustness and generalizability. Exploring the integration of domain-specific knowledge or additional modalities, such as functional MRI (fMRI) or diffusion tensor imaging (DTI), could extend the application of the model to broader clinical contexts. Moreover, conducting user studies and clinical trials to validate the model's performance in real-world scenarios would be crucial steps towards its adoption in clinical practice, potentially aiding radiologists in making more accurate diagnostic decisions.

## Data Availability

The data that support the findings of this study are available from the corresponding author upon reasonable request.

## Abbreviations

IQA: Image Quality Assessment
MRI: Magnetic Resonance Images
NR-IQA: No-Reference Image Quality Assessment
AI: Artificial Intelligence
GLRLM: Gray Level Run Length Matrix
SDBFN: Self-Evolving Deep Belief Fuzzy Neural Network
RMSE: Root Mean Square Error
SROCC: Spearman Rank Order Correlation Coefficient
KROCC: Kendall Rank Order Correlation Coefficient
MAE: Mean Absolute Error
NR: No-Reference
MRSA: Multi-Objective Reptile Search Algorithm
RPN: Region Proposal Network
TSPR: Two-Sided Pseudo Reference
LRE: Long Run Emphasis
RLN: Run Length Non-Uniformity
GLN: Gray Level Non-Uniformity
RP: Run Percentage
HGRE: High Gray Level Run Emphasis
ReLu: Rectifier Linear Unit
